# An α_2_-adrenoceptor agonist: Dexmedetomidine induces protective cardiomyocyte hypertrophy through mitochondrial-AMPK pathway

**DOI:** 10.7150/ijms.47598

**Published:** 2020-09-09

**Authors:** Xiaojian Weng, Hua Liu, Xiaodan Zhang, Qianqian Sun, Cheng Li, Minglu Gu, Yanyifang Xu, Shitong Li, Weiwei Li, Jianer Du

**Affiliations:** 1 Department of Anesthesiology and SICU, Xinhua Hospital, Shanghai, China.; 2 Department of Anesthesiology, The Ninth People's Hospital, Shanghai, China.; 3 Department of ICU, Shanghai General Hospital, Shanghai, China.; 4 Department of Anesthesiology, Shanghai General Hospital, School of Medicine, Shanghai Jiao Tong University, Shanghai, China.

**Keywords:** anesthesia, cardiac preconditioning, dexmedetomidine, cardiac hypertrophy, heart failure, mitochondria

## Abstract

**Aims:** Dexmedetomidine (Dex) as a highly selective α_2_-adrenoceptor agonist, was widely used anesthetic in perioperative settings, whether Dex induces cardiac hypertrophy during perioperative administration is unknown.

**Methods:** The effects of Dex on cardiac hypertrophy were explored using the transverse aortic constriction model and neonatal rat cardiomyocytes.

**Results:** We reported that Dex induces cardiomyocyte hypertrophy with activated ERK, AKT, PKC and inactivated AMPK in both wild-type mice and primary cultured rat cardiomyocytes. Additionally, pre-administration of Dex protects against transverse aortic constriction induced-heart failure in mice. We found that Dex up-regulates the activation of ERK, AKT, and PKC via suppression of AMPK activation in rat cardiomyocytes. However, suppression of mitochondrial coupling efficiency and membrane potential by FCCP blocks Dex induced AMPK inactivation as well as ERK, AKT, and PKC activation. All of these effects are blocked by the α_2_-adrenoceptor antagonist atipamezole.

**Conclusion:** The present study demonstrates Dex preconditioning induces cardiac hypertrophy that protects against heart failure through mitochondria-AMPK pathway in perioperative settings.

## Introduction

Considerable compelling clinical evidence and experiment results have suggested an important association between anesthetics and cardiac preconditioning in the perioperative setting 1, 2. Cardiac hypertrophy is a common preconditioning morphologic adaptation to various pharmacologic stimuli 3. However, whether an association exists between dexmedetomidine (Dex) preconditioning and cardiac hypertrophy is unknown.

Dex, which has been approved for anesthetic use in Europe since 2011, is a highly selective a_2_-adrenoceptor agonist with systemic sedative, analgesic, and anxiolytic effects that is suitable for short- and long-term sedation in intensive care settings 4, 5. In a recent study modeling sepsis, Dex was shown to attenuate systemic inflammation 6, whereas other studies demonstrated that Dex protects tissues against hypoxia-ischemia injury by suppressing apoptosis and improving adaptation to hypoxia 7-9. Other studies have shown that Dex enhances cell viability by decreasing production of harmful metabolites, such as reactive oxygen species (ROS) and lactate 10, 11. Collectively, these data suggest that Dex plays a protective role in various organs in perioperative settings via different molecular mechanisms. However, few reports have described the effect of Dex on various morphologic parameters.

Three highly homologous subtypes of the a_2_-adrenoceptor, a G protein-coupled receptor, have been described to date (a_2A_-, a_2B_-, and a_2C_-adrenergic receptor), and a fourth subtype, the a_2D_-adrenergic receptor, has been identified in several non-human species 12. Although a_2_-adrenoceptors are widely distributed in the central nervous system and play a role in presynaptic inhibition of neurotransmitter release, some animal experimental studies indicated that it is also expressed in the kidney, vasculature, and heart 11, 13, 14. Some G protein-coupled receptor agonists, such as endothelin-1 and phenylephrine, reportedly induce cardiomyocyte hypertrophy 15. However, whether Dex induces similar cardiac hypertrophy is unknown.

Cardiac hypertrophy is a regulated, adaptive morphologic response to excessive pressure or high output demand. Excessive pressure on the heart, as often seen in hypertension, causes pathologic cardiac hypertrophy; whereas long-term exercise causes physiologic cardiac hypertrophy, as seen in cases of athletic heart 3. Although both types of cardiac hypertrophy exhibit a similar phenotype of increased cardiac myocyte mass, they differ considerably in terms of molecular phenotype, cellular conductive pathway, and clinical consequences 16. The present study investigated the protective effect of Dex on cardiac hypertrophy, and involved *in vivo* experiments in mice and *in vitro* experiments using neonatal rat cardiomyocytes (NRCMs). Our results suggest that Dex-induced cardiac hypertrophy potentially protects the heart against transverse aortic constriction (TAC)-induced heart failure. Dex-induced hypertrophy of cardiac myocytes (cardiomyocytes) in both the *in vivo* and *in vitro* experiments via inactivation of AMPK-dependent activation of the ERK, AKT, and PKC signaling pathways. Furthermore, Dex-induced inactivation of AMPK is dependent on high mitochondrial coupling efficiency and membrane potential. Our present study for the first time investigated a novel effect of Dex on cardiomyocyte and provided a novel mechanism of Dex-induced the protective cardiac myocyte hypertrophy.

## Material and Methods

### Ethics approval

All experiments were approved by the Ethics Committee (Approval No.: XHEC-F-2017-076) and performed in accordance with relevant guidelines and regulations.

### Experimental animals

Male C57BL/6 mice were purchased from Shanghai Laboratory Animals Center (SLAC, Shanghai, China). Mice were provided free access to a normal chow diet and water under the conditions of the 23°C temperature and 50‑55% humidity with a 12-h light (08:00-20:00)/12-h dark (20:00-08:00) cycle.

### TAC model

Male mice (10~12 weeks old) weighing an average of 22-25 g were used to establish a TAC model, as reported previously 17. In brief, mice were anaesthetized by intraperitoneal injection of pentobarbital salt (50 mg/kg). The aortic arch was accessed via a left parasternal thoracotomy, and the thoracic aorta at the arch between the brachiocephalic trunk and left common carotid artery was surgically constricted using a 27-gauge needle to generate excessive pressure and progression to heart failure. Post-modeling, the Dex was administered for 2 consecutive weeks and observed mice survival for further 6-8 weeks.

### Cardiac echography

Mice were kept anaesthetized via inhalation of 2~3% isoflurane during the entire cardiac echography procedure. Transthoracic cardiac echography was performed using a Vevo 2100 Ultra-High-Frequency ultrasound device for small animal imaging (FujiFilm VisualSonics, Toronto, Canada) equipped with an MS 400 linear array transducer (18-38 MHz). M-mode recordings at the midventricular level were taken, and images were analyzed using dedicated software (Vevo 2100, version 1.4.0). Internal ventricular septum thickness, left ventricular wall thickness, and internal diameters were measured. Left ventricular volume, ejection fraction (EF), and percent left ventricular fractional shortening (%FS) were calculated from the M-mode measurements. All measurements and calculations were performed under double-blind conditions with regarding to either vehicle or Dex administration.

### NRCM culture

NRCMs were cultured as described in our previous report 11. In brief, 1~3-day-old neonatal Wistar rats were purchased from SLAC (Shanghai, China). The entire heart was removed from each rat and cut into small pieces, which were incubated in digestion solution (0.025% collagen type II, 0.06% trypsin, and 20 μg/ml DNase) at 37°C three times (15 min per time). Cardiomyocytes were separated using a Percoll gradient (45.5% + 58.5%) system. The collected cardiomyocytes were seeded for culture at 1×10^5^ cells per cm^2^ in Dulbecco's modified Eagle medium (Thermo Fisher Scientific, Inc., Waltham, MA, USA) containing 10% fetal bovine serum (Thermo Fisher Scientific) and incubated at 37°C in a 5% CO_2_/95% O_2_ atmosphere.

### Reagents use

Dex (Sigma; Merck Millipore, Darmstadt, Germany) was solubilized in sterile water. Based on the maximum Dex clinical plasma concentration (1.25 ng/ml), in *in vivo* experiments, Dex (0.7 μg/kg/h) was administered subcutaneously for 2 weeks via an osmotic pump (Alzet, Model 2002, Cupertino, CA, USA). For all *in vitro* experiments, cardiomyocytes were treated with Dex at 37°C for 24 h. For AMPK activation, cardiomyocytes were incubated with AICAR (1 mM) (Sigma, Shanghai, China) for 24 h simultaneously with Dex treatment. For suppression of mitochondrial coupling efficiency and membrane potential, cardiomycytes were incubated with FCCP (50 mM) (Sigma, Shanghai, China) for 24 h simultaneously with Dex treatment. For α_2_-adrenoceptor blocking, cardiomyocytes were pre-incubated with atipamezole (10 nM) (Sigma, Shanghai, China) for 30 min before Dex treatment.

### Mitochondrial TMRE assay and cellular flux analyzer

Cardiomyocytes mitochondrial coupling efficiency were calculated after measurement of cardiomyocyte OCR by Seahorse XF 24 extracellular analyzer (Agilent Technologies, Inc.) after Dex incubation for 24 h. Cardiomyocytes were cultured in collagen-coated 96-well dishes for TMRE assay (cat. no. ab113852; Abcam; Cambridge, UK) to detect mitochondrial membrane potential after Dex incubation for 24 h. The details have been reported in our previous study 11.

### Observation of live NRCMs and cellular area calculation

Cardiomyocytes were cultured in 12-well, collagen-coated multi-well dishes. Cardiomyocytes were observed and images were captured using a fluorescence microscope (Olympus FluoView™ FV1000; Olympus Corp., Tokyo, Japan). The area of cardiomyocytes was measured using ImageJ software (version 1.47; National Institutes of Health) from five randomly selected visual fields in each well. Three wells (15 visual fields) of cardiomyocytes were measured and calculated.

### [^3^H]-Leucine incorporation

NRCMs were pretreated with Dex for 24 h, washed with ice-cold sterile PBS three times after incubation with [^3^H]-leucine (1 μCi/ml) for 12 h, then incubated with ice-cold trichloroacetic acid (5% [v/v]) at 4°C for 1 h. After another wash with ice-cold, sterile PBS, NRCMs were suspended in 0.1 mol/l NaOH. Radioactivity was measured using a Beckman LS6500 liquid scintillation counter (Beckman Instrument, Inc. Fullerton, CA, USA).

### Cellular immunofluorescence staining

Cardiomyocytes were cultured in 4-well, multi-chamber glass slides (MatTek Corp., Tokyo, Japan). Following Dex treatment for 24 h, cells were washed with sterile PBS three times (5 min each) and fixed in cold acetone for 20 min, then washed with TBS three times. Following blocking with BSA (Sigma; Merck Millipore) for 1 h at room temperature (24-26°C), immunofluorescence staining was performed using an anti-Myh6 antibody (goat pAb; 1:600; sc-168676; Santa Cruz Biotechnology, Inc.) and donkey anti-goat IgG (red) (AlexaFluor^®^ 488, Abcam, Shanghai, China). Counterstaining with fluorescence mounting medium containing DAPI (blue; Thermo Fisher Scientific, Inc.) was performed to visualize normal nuclei. Sections were observed using a fluorescence microscope (Olympus FluoView™ FV1000; Olympus Corp.).

### Histologic analysis

Mouse heart tissue was fixed in 4% formaldehyde (Sinopharm Chemical Reagent Co., Ltd, Shanghai, China) at room temperature for more than 12 h, then embedded in paraffin after complete dehydration using ethyl alcohol. Blocks were cut into 4-μm-thick slices, air-dried, dewaxed in xylene, and stained with hematoxylin and eosin (Sinopharm Chemical Reagent Co., Ltd., Shanghai, China). Heart tissue images were captured using a fluorescence microscope (Olympus FluoView™ FV1000; Olympus Corp.) and light-field observation model.

### RT-qPCR

Total RNA extraction from cultured cardiomyocytes or heart tissue, cDNA synthesis, and real-time PCR amplification were performed according to routine methods described in our previous report 11. Results were normalized to expression of the 18s rRNA gene. The primers used in the present study were reported previously 17 and are shown in Supplementary Table 1.

### Western blot analysis

Whole protein from NRCMs or mouse heart tissue was extracted as previously reported 11. Intercellular signaling was analyzed using a Pathscan Intercellular Signaling Array kit (#7323 and #12856; Cell Signaling Technology, Inc.) following the protocol provided, and routine western blotting procedures were performed according to our previous report 11. The primary antibodies used were as follows: anti-ANP (mouse mAb; 1:300; sc-515701; Santa Cruz Biotechnology, Inc.), anti-BNP (rabbit pAb; 1:500; ab19645; Abcam), anti-MYH7 (mouse mAb; 1:300; sc-53089; Santa Cruz Biotechnology, Inc.), anti-Erk1/2 (rabbit mAb; 1:1000; #9102), anti-phospho-Erk1/2 (Thr202/Thr204) (rabbit mAb; 1:1000; #9102), anti-Akt (rabbit mAb; 1:1000; #9272), anti-phospho-Akt (Ser473) (rabbit mAb; 1:1000; #4060), anti-PKC (rabbit mAb; 1:1000; #2056), anti-phospho-PKC pan (Thr514) (rabbit mAb; 1:1000; #9379), anti-AMPK (rabbit mAb; 1:1000; #5831), anti-phospho-AMPK (Thr172) (rabbit mAb; 1:1000; #2535), and anti-GAPDH (rabbit mAb; 1:2000; #2118;Cell Signaling Technology, Inc.). The secondary antibodies used were horseradish peroxidase (HRP) conjugated (GE Healthcare Life Sciences, Beijing, China): anti-mouse IgG, HRP-conjugated whole Ab sheep (NA931) or anti-rabbit IgG, HRP-linked whole Ab donkey (NA934).

### Statistical analysis

All experiments were repeated two or three times. All results are reported as the mean ± standard error of the mean. The normality of distribution was analyzed using the D'Agostino-Pearson omnibus normality test (version 6.0; GraphPad Software, Inc., La Jolla, CA, USA). Comparisons between two groups were analyzed using the Student's *t*‑test. Multiple comparisons between groups were performed using one‑way analysis of variance with Tukey's multiple comparisons test. Mouse survival was analyzed using the Kaplan-Meier log-rank test. Both one-way analysis of variance and Kaplan-Meier tests were performed using GraphPad Prism software (version 6.0). A *P* value of <0.05 was considered indicative of a statistically significant difference.

## Results

### Dex induces cardiac hypertrophy and protects against heart failure in mice

**Figure 1:**
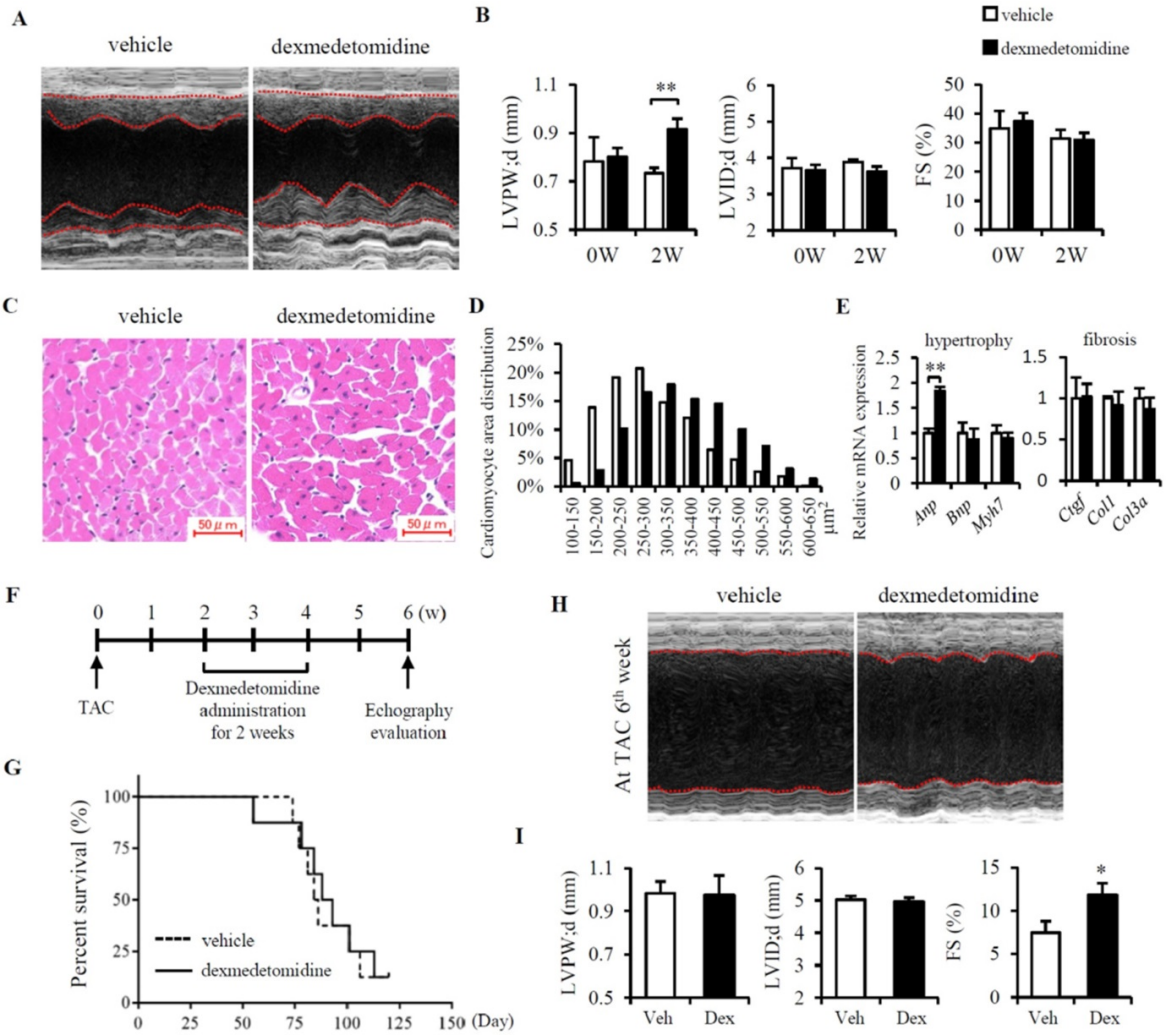
** Dex induces cardiomyocyte hypertrophy and protects against heart failure in mice.** (**A**) Representative cardiac echographs of vehicle- and Dex-treated mice. (**B**) LVPW, LVID, and FS% values determined from cardiac echographs shown in panel (A). (**C**) Representative images of hematoxylin and eosin-stained heart tissues from mice administered vehicle or Dex (scale bar: 50 μm). (**D**) Distribution of cardiomyocyte area in heart tissues from mice shown in panel (C). (**E**) Relative expression of mRNAs for cardiomyocyte hypertrophy markers (*Anp*, *Bnp*, *Myh7)* and cardiac fibrosis markers (*Ctgf*, *Col1*, *Col3a*) in vehicle and Dex-treated mice. Values for vehicle were set to 1. (**F**) Schedule of Dex administration in mice with TAC. (**G**) Kaplan-Meier survival curves for vehicle- and Dex-treated mice with TAC. (**H**) Representative cardiac echographs in vehicle- and Dex-treated mice with TAC. (**I**) LVPW, LVID, and FS% values were determined from cardiac echographs of H. Data are mean ± SEM (n=5 per group in each group. * *P*<0.05; ** *P*<0.01).

Firstly, to know whether Dex induces cardiac hypertrophy *in vivo*, mice were administered Dex subcutaneously for 2 weeks, after which cardiac echography was performed. We found that Dex significantly increased cardiac left ventricular posterior wall thickness (LVPW) but had no effect on left ventricular internal dimension (LVID) or %FS (Fig. 1A, B and Table 1). Consistently, we also observed enlarged cardiomyocytes in mice administered Dex compared to mice that received vehicle (Fig. 1C and D), accompanied with a significant increase in *Anp* mRNA levels (Fig. 1E). Dex did not increase the expression of several cardiac fibrosis markers (*Ctgf*, *Col1*, and *Col3a*) (Fig. 1E), indicating that Dex induces cardiac myocyte hypertrophy without cardiac fibrosis.

After this, we evaluated whether Dex-induced cardiac myocyte hypertrophy is beneficial or detrimental by using TAC model in mice. At 2 weeks after the TAC operation, the mice were administered Dex subcutaneously for 2 weeks. After another 2 weeks, cardiac function was evaluated using cardiac echography (Fig. 1F). Although we didn't find any difference in Mice survival between vehicle and Dex administered mice (Fig. 1G), mice administered Dex exhibited an increase in %FS but no changes in LVPW and LVID compared with mice administered with vehicle (Fig. 1H, I and Table 2). To further determine whether this attenuation in the severity of heart failure induced by Dex was associated with attenuated cardiac fibrosis, we evaluated the degree of cardiac fibrosis in the two groups of mice by examining the levels of several fibrosis markers (*Ctgf*, *Col1*, and *Col3a*) using RT-PCR and heart Masson trichrome staining. However, no significant differences were observed between mice administered Dex and vehicle (data not shown).

In total, TAC induced LVPW increased from 0.73 mm to 0.98mm (increased about 34%). In Dex group, after TAC, LVPW increased from 0.92 mm to 0.98 mm (increased only 6.5%) (Fig. 1B, I). This might indicates that Dex preconditioning helps the heart resist hypertrophy induced by TAC surgery. Additionally, Dex preconditioning did not affect heart FS% compared to the vehicle group (31.0% vs. 31.5%), but Dex preconditioning did prevent a decrease in FS% compared to the vehicle group (11.85% vs. 7.44%) (Fig. 1B and I). Dex preconditioning induces protective hypertrophy, providing protection against TAC-induced heart failure that might be a beneficial morphologic change.

### Dex induces NRCMs growth with high protein synthesis

**Figure 2:**
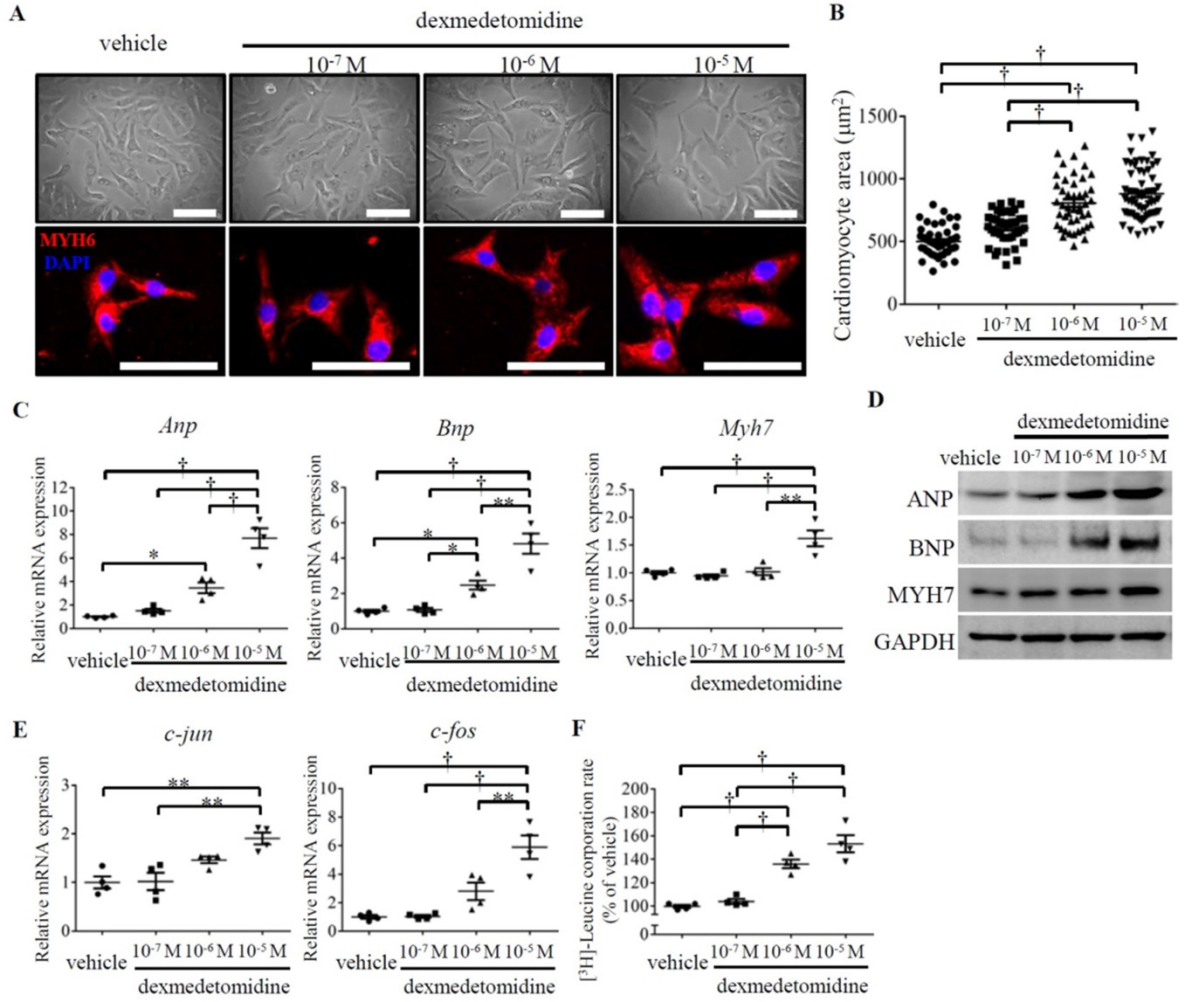
** Dex induces NRCMs enlargement with up-regulated protein synthesis.** (**A**) Representative live NRCMs (top) observed by light microscopy and MYH6-stained NRCMs (bottom) observed by fluorescence microscopy in control cells and cells treated with three concentrations (10^-7^, 10^-6^, and 10^-5^ M) of Dex (red: MYH6-stained NRCMs; blue: DAPI-stained NRCM nuclei. Scale bars: 100 μm). (**B**) Cardiomyocyte area of NRCMs shown in panel (A).(**C and E**) Relative expression of *Anp*, *Bnp*, *Myh7* (C) and *c-jun* and *c-fos* (E) mRNAs in NRCMs treated with vehicle and three concentrations (10^-7^, 10^-6^, and 10^-5^ M) of Dex. Values for the vehicle group were set to 1. (**D**) Representative western blot results for expression of ANP, BNP, and MYH7 in vehicle and Dex-treated cardiomyocytes. GAPDH was used as a loading control. (**F**) [^3^H]-Leucine incorporation rate in vehicle and Dex-treated cardiomyocytes. Value for the vehicle group was set to 100%. Data are mean ± SEM (* *P*<0.05; ** *P*<0.01; † *p*<0.001).

To investigate the primary effect of Dex on cardiomyocyte hypertrophy, we prepared primary cultured NRCMs and exposed them to Dex at three different doses (10^-7^, 10^-6^, and 10^-5^ M). After 24 h of Dex exposure on NRCMs, the morphology of live and MYH6-stained NRCMs was observed under an optical or fluorescence microscope, and the area of the NRCMs was calculated. Dex-treated NRCMs were much larger than NRCMs in the vehicle treated group in a dose dependent manner (Fig. 2A and B). To further confirm that the observed increases in NRCMs size was induced by Dex, we evaluated cardiomyocyte hypertrophy markers using RT-PCR and western blotting. We found that levels of *Anp* and *Bnp* were significantly up-regulated in cells treated with both the 10^-6^ and 10^-5^ M doses of Dex, whereas levels of *Myh7* were significantly up-regulated only in cells treated with 10^-5^ M Dex (Fig. 2C and D).

Proto-oncogenes are known to regulate cell proliferation and growth 18. To evaluate whether Dex induces NRCMs growth, we investigated the expression of two proto-oncogenes: *c-jun* and *c-fos.* The expression of both proto-oncogenes was significantly up-regulated in NRCMs treated with 10^-5^ M Dex compared with vehicle treated NRCMs (Fig. 2E). As cardiomyocyte hypertrophy is typically associated with enhanced protein synthesis 19, we next investigated protein synthesis in Dex-treated NRCMs. The [^3^H]-leucine incorporation rate increased significantly, by 138% and 150%, in NRCMs treated with 10^-6^ M and 10^-5^ M Dex, respectively (Fig. 2F). Collectively, these results indicate that Dex promotes NRCM hypertrophy, as evidenced by increases in cell area, cell growth marker expression, and protein synthesis.

However, at 10^-7^ M, Dex did not affect the expression of cardiomyocyte hypertrophy markers (*Anp*, *Bnp*, and *Myh7*), cell growth markers (*c-jun* and *c-fos*), or protein synthesis (Fig. 2C-F), which could explain why Dex treatment at 10^-7^ M did not promote NRCMs hypertrophy (Fig. 2A and B).

### ERK, AKT, PKC, and AMPK are involved in Dex-induced NRCMs hypertrophy

**Figure 3:**
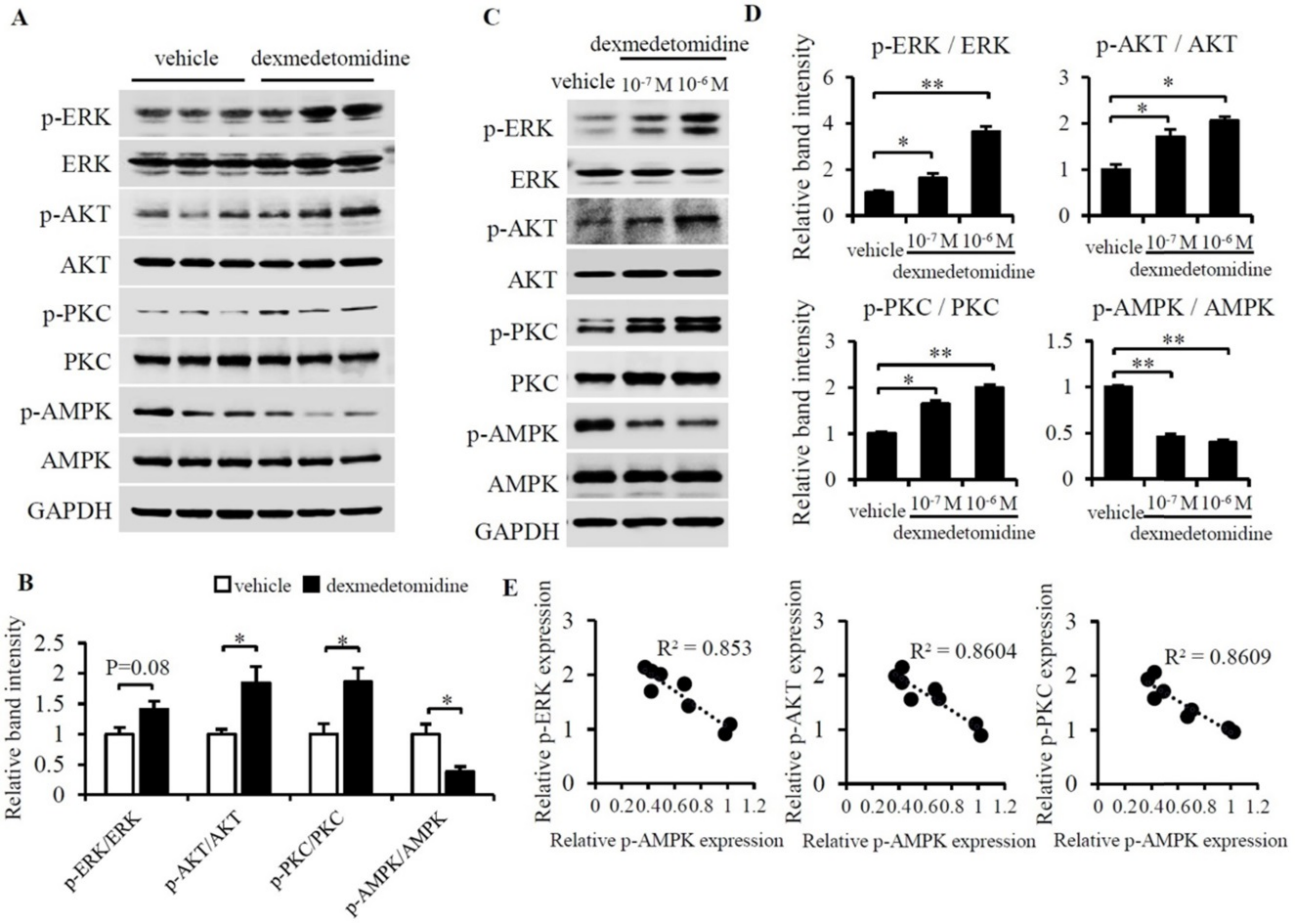
** Dex activates ERK, AKT, PKC and inactivates AMPK in mice heart tissue and NRCMs.** (**A**) Representative western blots of expression of ERK, AKT, PKC, and AMPK and their phosphorylated forms in vehicle- and Dex-treated mice heart tissue. GAPDH was used as a loading control. (**B**) Relative quantification of the ratios of p-ERK/ERK, p-AKT/AKT, p-PKC/PKC, and p-AMPK/AMPK as determined from western blot bands shown in panel (A). Values of vehicle group were set to 1. (**C**) Representative western blots of expression of ERK, AKT, PKC, and AMPK and their phosphorylated forms. GAPDH was used as a loading control. (**D**) Relative quantification of the ratios of p-ERK/ERK, p-AKT/AKT, p-PKC/PKC, and p-AMPK/AMPK as determined from western blot bands in panel (C). Values for the vehicle group were set to 1. (**E**) Linear relationship between p-AMPK and p-ERK, p-AKT, or p-PKC in each NRCM group. Data are mean ± SEM (* *P*<0.05; ** *P*<0.01).

We then sought to determine what signaling pathway mediates cardiomyocyte hypertrophy induced by Dex. We examined lots of key protein by using western blotting and finally we found phosphorylation of ERK, AKT, and PKC were up-regulated whereas phosphorylation of AMPK was down-regulated in heart tissue of mice administered Dex compared to tissue from mice administered vehicle (Fig. 3A and B). These results indicate that the inactivation of AMPK and activation of ERK, AKT and PKC maybe involved in Dex promoted cardiac hypertrophy. We furtherly investigated these proteins in NRCMs, we found the same results showed inactivated AMPK and activated ERK, AKT and PKC in NRCMs after Dex treatment for 24 h (Fig. 3C and D).

To further examine the potential protein involved in Dex-induced cardiomyocytes hypertrophy, we investigated cyclopedic conductive signaling proteins using intracellular signaling pathway array analysis (Supplementary Figure 1), which included 18 protein kinases. We found that the expression of 7 of the protein kinases changed in response to Dex. Of these kinases, except the most changed four proteins (p-ERK1/2, p-AKT, p-PKC and p-AMPK), we also found the expression of p-PTEN, p-mTOR and p-Stat3 [s727] were also increased in Dex-treated NRCMs compared to control NRCMs (Supplementary Figure 1). This analysis indicated p-PTEN, p-mTOR and p-Stat3 [s727] also play some potential role on Dex-induced cardiomyocyte hypertrophy.

As it was reported that overexpression of either ERK1/2, AKT, or PKC can cause compensated or concentric cardiac hypertrophy 20-22 and AMPK activation can prevent cardiac hypertrophy 23, we hypothesized that activation of ERK1/2, AKT, and PKC and inactivation of AMPK mainly mediates Dex-induced cardiomyocyte hypertrophy.

### Dex-mediated up-regulation of p-ERK, p-AKT, and p-PKC is dependent on inactivation of p-AMPK

**Figure 4:**
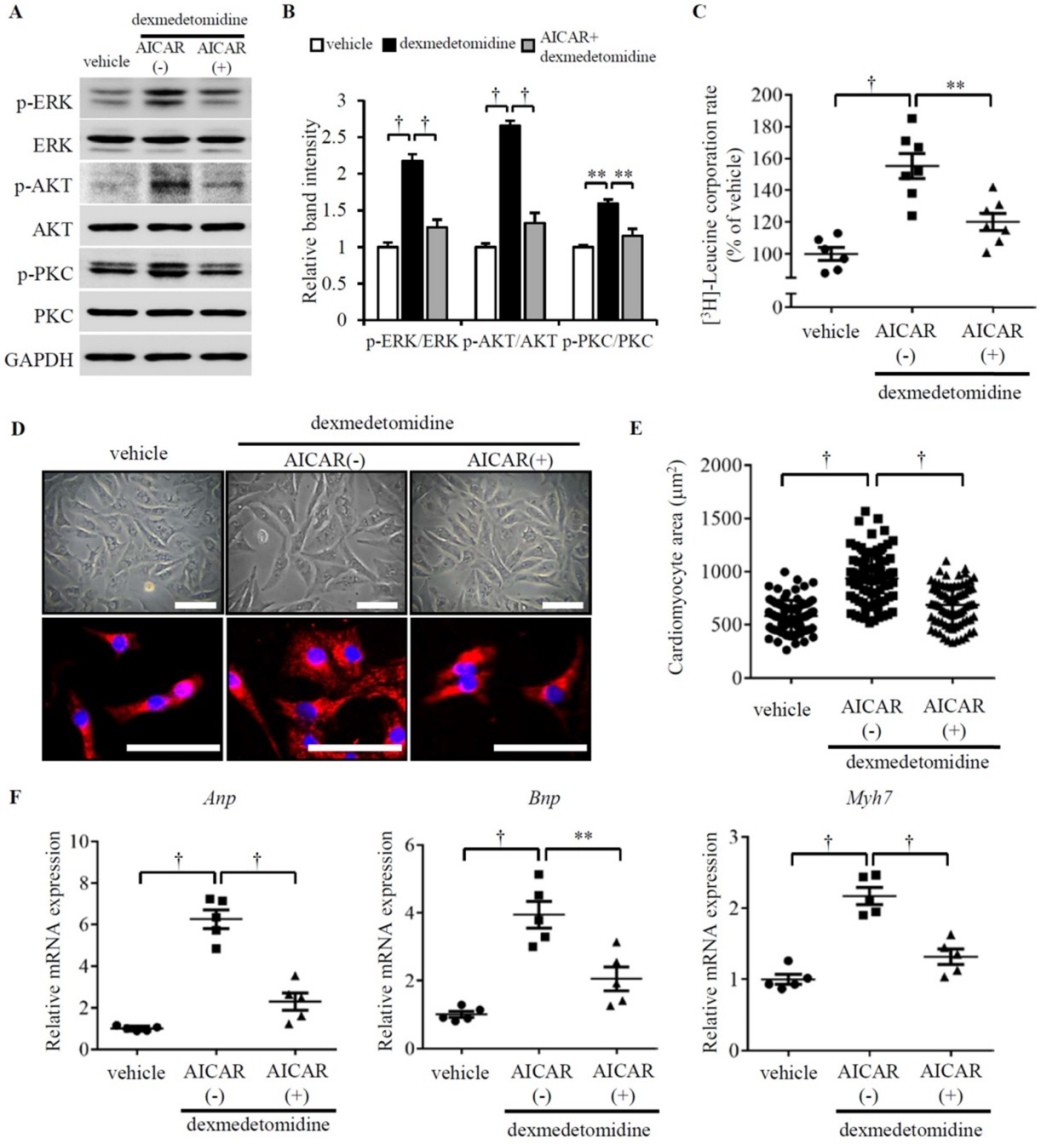
** Dex-induced NRCMs hypertrophy depends on the inactivation of p-AMPK.** (**A**) Representative western blots of expression of ERK, AKT, and PKC and their phosphorylated forms in control and Dex-treated cells with and without AICAR pre-incubation. GAPDH was used as a loading control. (**B**) Relative quantification of the ratios of p-ERK/ERK, p-AKT/AKT, and p-PKC/PKC as determined from western blot bands shown in panel (A). Values of vehicle group were set to 1. (**C**) [^3^H]-Leucine incorporation rate in vehicle and Dex-treated NRCMs with and without AICAR pre-incubation. Value of vehicle group was set to 100%. (**D**) Representative live NRCMs (top) and MYH6-stained NRCMs (bottom) in vehicle and Dex-treated groups with and without AICAR (AMPK activator) pre-incubation (red: MYH6-stained NRCMs; blue: DAPI-stained NRCM nuclei. Scale bars: 100 μm). (**E**) Area of NRCMs shown in panel (D). (**F**) Relative expression of *Anp*, *Bnp*, and *Myh7* mRNAs in vehicle and Dex-treated cardiomyocytes with and without AICAR pre-incubation. Values of vehicle group were set to 1. Data are mean ± SEM (n=5-7 per group in C and F, n=3-4 per group in B; ** *P*<0.01; † *P*<0.001).

As we observed that ERK, AKT, and PKC were up-regulated, while AMPK was down-regulated, we sought to determine whether there is a relationship between the reversed changes in Dex-treated cardiomyocytes. Reports have indicated that AMPK activation suppresses cardiac protein synthesis and hypertrophy via inhibition of ERK, AKT, and PKC in cardiomyocytes 24-26 and the AMPK activator 5-aminoimidazole-4-carboxamide riboside (AICAR) inhibits activation of AKT in neurocytes 27. We indeed found down-regulated p-AMPK is linear related with up-regulated p-ERK, p-AKT, and p-PKC (Fig. 3E). These reports and observations suggest a likelihood that Dex-induced up-regulation of p-ERK, p-AKT, and p-PKC may be associated with inactivation of AMPK. To test this hypothesis, we used AICAR to activate AMPK and investigated whether AMPK activation blocks Dex-induced NRCM hypertrophy. Interestingly, as we hypothesized, AICAR blocks Dex-induced activation of ERK, AKT, and PKC (Fig. 4A and B) as well as Dex-induced protein synthesis (Fig. 4C) in NRCMs. Additionally, AICAR-preconditioned NRCMs exhibited lower cardiomyocyte area compared to vehicle preconditioned NRCMs treated with Dex (Fig. 4D and E). To confirm this result, we also investigated the expression of cardiomyocyte hypertrophy markers. The expression of *Anp*, *Bnp*, and *Myh7* mRNAs did not increase after Dex treatment in AICAR-preconditioned NRCMs, compared with vehicle-conditioned NRCMs (Fig. 4F). Collectively, these results suggest that AMPK activation blocks ERK, AKT, and PKC activation, protein synthesis, and cardiomyocyte hypertrophy, indicating that AMPK inactivation plays a crucial role.

### Dex induces inactivation of AMPK is dependent on elevated mitochondrial coupling efficiency and membrane potential

**Figure 5:**
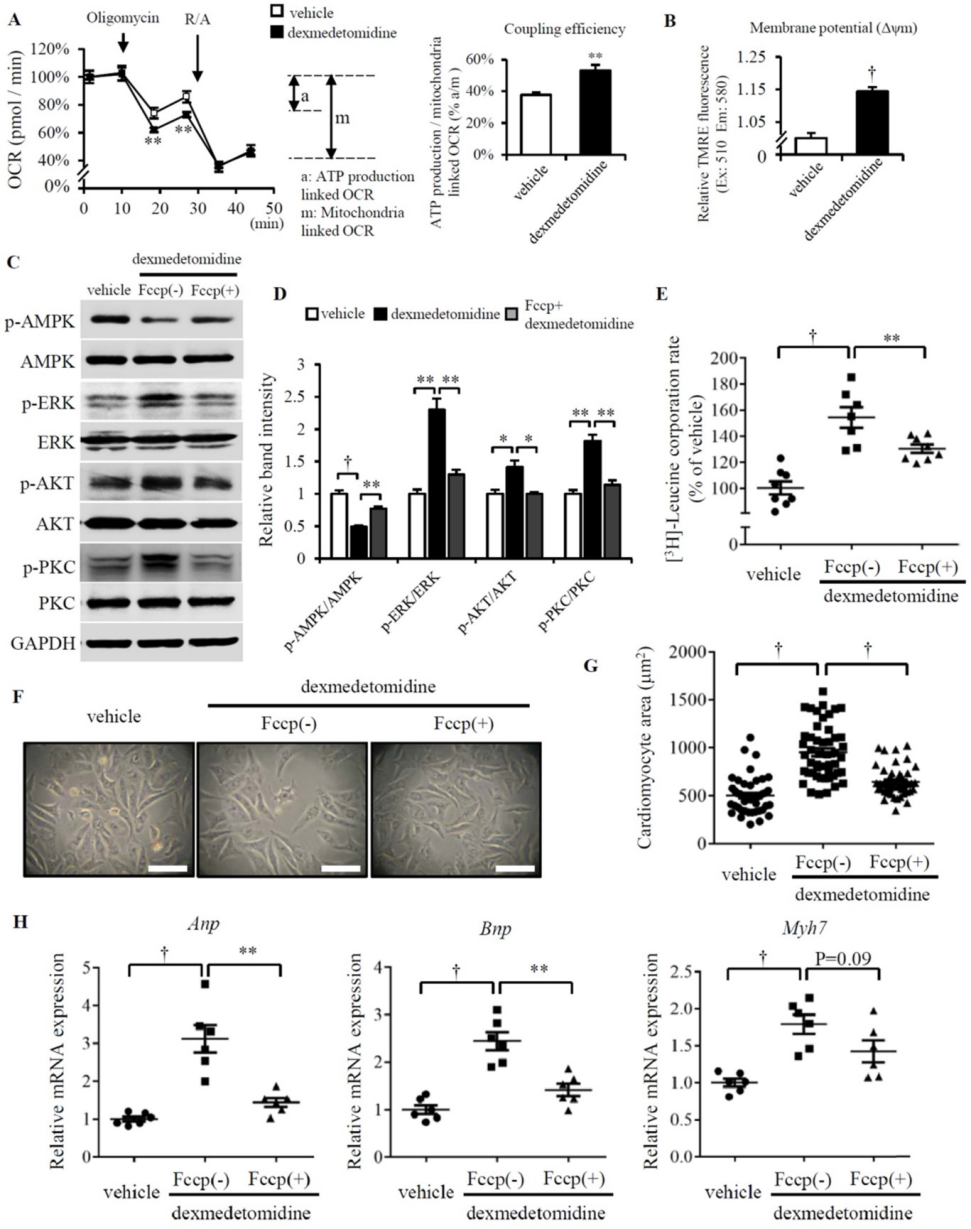
** Increased mitochondrial function facilities Dex-induced NRCMs hypertrophy.** (**A**) Flux analysis indicates cellular OCR change in mitochondrial stress (left) and the calculated coupling efficiency determined by ATP production-/mitochondrial-linked OCR (right) in NRCMs incubated with or without Dex. (**B**) Mitochondrial membrane potential determined by mitochondrial TMRE assay in NRCMs incubated with or without Dex. (**C**) Representative western blots of expression of ERK, AKT, PKC, and AMPK and their phosphorylated forms in vehicle and Dex-treated groups with and without FCCP pre-incubation. GAPDH was used as a loading control. (**D**) Relative quantification of the ratios of p-ERK/ERK, p-AKT/AKT, p-PKC/PKC, and p-AMPK/AMPK as determined from western blot bands shown in panel (C). Values of vehicle were set to 1. (**E**) [^3^H]-Leucine incorporation rate in vehicle and Dex-treated NRCMs with and without FCCP pre-incubation. Value of vehicle group was set to 100%. (**F**) Representative live NRCMs in vehicle and Dex-treated groups with and without FCCP pre-incubation (Scale bars: 100 μm). (**G**) Area of NRCMs shown in panel (F). (**H**) Relative expression of *Anp*, *Bnp*, *Myh7* mRNAs in vehicle and Dex-treated groups with and without FCCP pre-incubation. Values of vehicle group were set to 1. Data are mean ± SEM (n=10-12 per group in A and B, n=3-4 per group in D, n=6-8 per group in E and H; * *P*<0.05; ** *P*<0.01; † *p*<0.001).

We next asked why AMPK is inactivated after Dex treatment. In our previous study, we reported Dex enhanced NRCMs mitochondrial coupling efficiency and membrane potential (Δψm) 11. Other report also showed FCCP induced mitochondrial uncoupling increased AMPK activation 28. So, these made us think about whether the inactivation of AMPK induced by Dex is resulted from the enhanced coupling efficiency and mitochondrial Δψm. Before examining this hypothesis, we firstly confirmed Dex elevates mitochondrial coupling efficiency and Δψm (Fig. 5A and B), the similar result with our previous report 11. FCCP, a well-known mitochondrial potent uncoupler, suppresses mitochondrial coupling efficiency and Δψm 29, 30. Next, we used FCCP to inhibit NRCMs mitochondrial coupling efficiency and Δψm, then we found FCCP increases AMPK activation and decreases ERK, AKT, PKC activation in Dex incubated NRCMs compared to that treated without FCCP (Fig. 5C and D). We also found the protein synthesis is blocked in Dex incubated NRCMs treated with FCCP compared with that treated without FCCP (Fig. 5E). Furtherly, paralleled with these results, FCCP suppresses the increased NRCMs area induced by Dex (Fig. 5F and G) and suppresses the increased *mRNA* expression (*Anp*, *Bnp* and *Myh7*) induced by Dex (Fig. 5H). Taken together, these results suggest the inhibition of mitochondrial coupling efficiency and Δψm facilitates the activation of AMPK, inactivation of ERK, AKT and PKC, as well as suppresses the following NRCMs protein synthesis and NRCMs hypertrophy.

### Dex induces α_2_-adrenergic receptor-mediated cardiac myocyte hypertrophy

**Figure 6:**
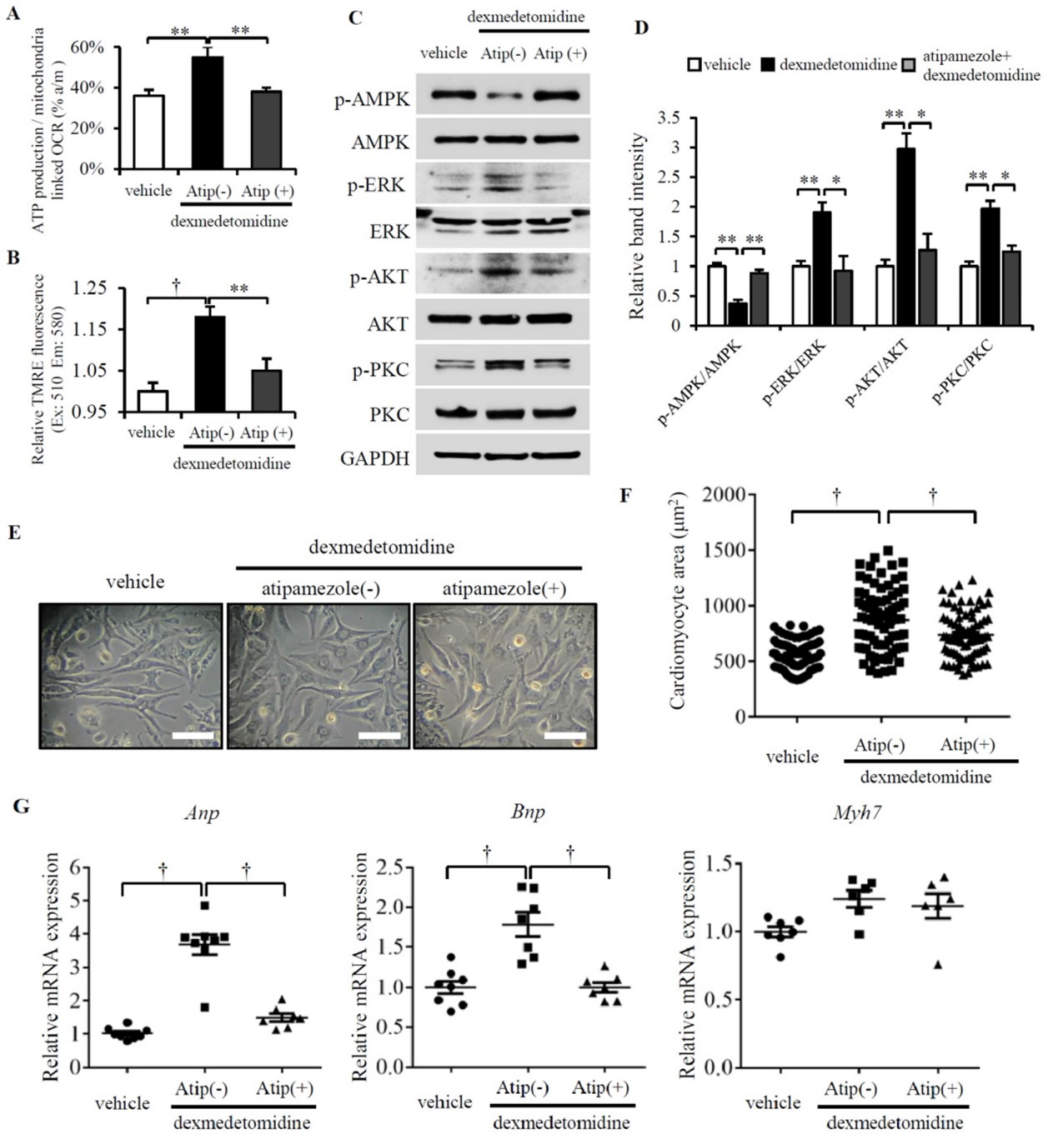
** Atipamezole blocks Dex-induced NRCMs hypertrophy.** (**A and B**) Mitochondrial coupling efficiency determined by ATP production-/mitochondrial-linked OCR (A) and mitochondrial membrane potential (B) in vehicle or Dex-treated NRCMs with or without preconditioning of atipamezole. (**C**) Representative western blots of expression of ERK, AKT, PKC, and AMPK and their phosphorylated forms in vehicle and Dex-treated groups with and without atipamezole pre-incubation. GAPDH was used as a loading control. (**D**) Relative quantification of the ratios of p-ERK/ERK, p-AKT/AKT, p-PKC/PKC, and p-AMPK/AMPK as determined from western blot bands shown in panel (C). Values of vehicles were set to 1. (**E**) Representative live NRCMs in vehicle and Dex-treated groups with and without atipamezole pre-incubation (Scale bars: 100 μm). (**F**) Area of NRCMs shown in panel (E). (**G**) Relative expression of *Anp*, *Bnp*, *Myh7* mRNAs in vehicle and Dex-treated groups with and without atipamezole pre-incubation. Values of vehicle group were set to 1. Data are mean ± SEM (n=6-8 per group in (C), n=3-4 per group in (E). * *P*<0.05; ** *P*<0.01; † *p*<0.001).

Although Dex is an α_2_-adrenergic receptor agonist, whether Dex-induced cardiomyocyte hypertrophy is mediated by α_2_-adrenergic receptors is unknown. To resolve this question, we pretreated cardiomyocytes with atipamezole (α_2_-adrenergic receptor antagonist) for 30 min prior to Dex treatment. After 24 h, we examined mitochondrial coupling efficiency, Δψm and found both are decreased in NRCMs pretreated with atipamezole (Fig. 6A and B). We next further investigated the activation of AMPK, ERK, AKT and PKC. We found Dex increased the phosphorylation of ERK, AKT, and PKC and decreased the phosphorylation of AMPK; however, atipamezole blocked these Dex-induced changes completely (Fig. 6C and D). Next, we quantified the cardiomyocyte area and the involved *Anp* and *Bnp* mRNA expression. However, atipamezole-pretreated cardiomyocytes exhibited less hypertrophy, and *Anp* and *Bnp* mRNA expression was completely blocked (Fig. 6E-G). These results demonstrate that α_2_-adrenergic receptors mediate Dex-induced cardiomyocyte hypertrophy.

## Discussion

**Figure 7:**
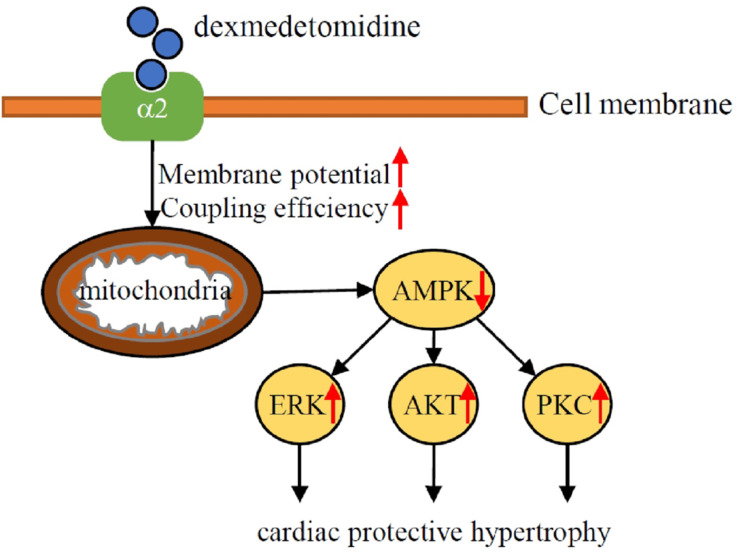
Schematic illustration of the results of the present study indicating that Dex preconditioning elevates cardiomyocytes mitochondrial coupling efficiency and membrane potential, which induces the inactivation of AMPK via α2-adrenergic receptor. Down-regulated AMPK further facilitates activation of ERK, AKT, PKC signaling and lead to cardiac hypertrophy, which protects heart against heart failure.

To the best of our knowledge, the results of the present study was the first to demonstrate that Dex induces a protective hypertrophy in mouse heart and NRCMs via α_2_-adrenergic receptor- and mitochondria- mediated inactivation of AMPK and activation of the ERK, AKT, and PKC pathways. The results of this study found Dex-induced activation of the ERK, AKT, and PKC pathways depends on the inactivation of AMPK and elevated mitochondrial coupling efficiency and membrane potential. When we administered Dex to mice with TAC, although we found that Dex don't affect heart failure related survival, Dex protects against TAC-induced heart failure but had no effect on survival. These results suggest that cardiac preconditioning with Dex in the perioperative setting has a protective effect against cardiac damage (Fig. 7).

Cardiac hypertrophy is a complex adaptation process involving either extracellular or intracellular signaling circumstance towards to various drugs or physiological requirement. Activation of AMPK inhibits protein synthesis and cardiac hypertrophy 23, 24; conversely, activation of either ERK, AKT or PKC up-regulates protein synthesis and induces cardiac hypertrophy 20-22. Furthermore, the effect of AMPK activation was dependent on the down-regulation of ERK, AKT, or PKC 25-27. These previous reports suggest that suppression of AMPK activation accelerates ERK, AKT, and PKC activation. Although one study suggested that Dex preconditioning induces the expression of pro-survival kinases in heart tissue, including ERK and AKT 14, the results were not significant, and no mechanism leading to up-regulation of these pro-survival kinases was proposed. The present study, however, demonstrated that Dex up-regulates the phosphorylation of ERK, AKT, and PKC in an AMPK dephosphorylation-dependent manner.

Reports indicate that ERK, AKT, and PKC play an important role not only in cardiac hypertrophy, but also cell survival 31, 32. Previous reports indicated that Dex preconditioning increases the expression of pro-survival kinases and attenuates ischemia/reperfusion injury in rat heart and brain 8, 14. In these previous studies, the authors only reported the potential pro-survival role of these kinases but ignored their role in morphologic adaptation. We found that administration of Dex for 2 weeks induces cardiac hypertrophy in mice, which has a protective role against TAC-induced heart failure over the short term but does not affect TAC-associated mortality. This result led us to consider that although Dex-induced cardiac hypertrophy is temporary, this short-term effect explains the importance of preconditioning in the perioperative setting.

Exercise is known to induce physiologic cardiac hypertrophy, which protects against heart failure via a very complex mechanism involving the activation of ERK, AKT, and PKC 33-35. Interestingly, we found that Dex also induces protective cardiac hypertrophy via activation of ERK, AKT, and PKC, which led us to consider that Dex-induced cardiac hypertrophy may be more similar with physiologic hypertrophy than pathologic hypertrophy. In 1984, it was reported that α_2_-adrenergic receptors are activated during exercise 36. As the results of our present study demonstrated that Dex preconditioning induces physiologic cardiac hypertrophy involving α_2_-adrenergic receptors, we hypothesized that exercise-induced α_2_-adrenergic receptor activation with subsequent activation of ERK, AKT, and PKC is similar with the condition induced by Dex. Moreover, it was reported that failing hearts were accompanied with mitochondria dysfunction and reduced oxygen consumption and exercise enhances mitochondrial function 37, 38. This similarity could explain why the physiologic-like cardiac hypertrophy induced by Dex protects against heart failure. Contrary to Dex, several studies reported that exercise increased the activation of AMPK in skeletal muscles 39, 40, whereas, Grassi *et al.* did not support this statement 41 in cardiac functions, hence future studies are needed to compare the protection mechanism between Dex and exercise in heart failure.

Our previous study demonstrated that Dex (100 nM) reduces ROS sensitivity in NRCM mitochondria via downregulation of mitochondrial respiratory complexes I, II, and III and upregulation of Bcl2 protein expression, which protects NRCMs against ROS-induced apoptosis, thus enhancing NRCM survival 11. However, the mechanism by which Dex induces this mitochondrial phenotype remains to be elucidated. A number of reports have demonstrated that ERK, AKT, and PKC are closely associated with mitochondrial function. For example, phosphorylated ERK translocates to the mitochondria, where it regulates Bcl2 expression and decreases mitochondrial cytochrome C release 42, 43. AKT accumulation in mitochondria of cardiomyocytes protects the cells by preventing opening of the mitochondrial permeability transition pore 44, 45, whereas PKC activates mitochondrial ATP-sensitive K^+^ channels, thus protecting against ischemic injury 46. All of these reports describe ERK, AKT, or PCK activation in mitochondria, which could explain how Dex regulates sensitivity to ROS and Bcl2 expression in NRCM mitochondria. However, very interestingly, we demonstrated mitochondrial coupling efficiency and membrane potential, induced by Dex, are necessary for AMPK inactivation and ERK, AKT, or PCK activation, which are partially consistent with the previous report 28. This suggests Dex induced preferable mitochondrial change closely linked with the inactivation of AMPK.

For limitation, we hypothesized that AMPK inactivation is essential for the regulation of cardiomyocyte hypertrophy induced by Dex. However, we failed to prove whether AMPK inhibition could induce cardiomyocyte hypertrophy in this study. In addition, we noted that Dex increases Anp in heart but increases Anp, Bnp, Myh7 in cardiomyocytes. This discrepancy may be caused by the complicated atmosphere of tissues or due to species difference, since the cardiomyocytes were isolated from rats and the *in vivo* analysis were performed on mice. Moreover, clinical data are needed to validate the findings observed in animals and cells.

In summary, the *in vitro* and *in vivo* results of the present study demonstrate for the first time that preconditioning with Dex induces cardiomyocyte hypertrophy that protects against heart failure via α_2_-adrenergic receptor- and mitochondria- mediated AMPK dephosphorylation and ERK, AKT, and PKC phosphorylation. Our demonstration of Dex-induced morphologic cardiac changes in mice provides novel important evidence of the beneficial effects of Dex preconditioning in perioperative settings.

## Supplementary Material

Supplementary figures and tables.

## Figures and Tables

**Table 1:** Echography parameters of wild-type mice administered either Dex or vehicle

Measurement	Units	Administration group		*P* value
		Vehicle	Dexmedetomidine	
**IVST**				
diastole	mm	0.87±0.04	0.83±0.05	0.59
systole	mm	1.31±0.10	1.26±0.06	0.69
**LVID**				
diastole	mm	3.89±0.07	3.62±0.14	0.12
systole	mm	2.67±0.16	2.50±0.14	0.44
**LVPW**				
diastole	mm	0.73±0.02	0.92±0.04	0.0032
systole	mm	1.15±0.06	1.20±0.05	0.50
**LV volume**				
diastole	μL	65.6±2.9	55.9±5.2	0.14
systole	μL	27.1±4.0	22.9±3.1	0.43
**EF**	%	59.4±4.6	59.3±3.4	0.98
**FS**	%	31.5±3.1	31.0±2.3	0.91

IVST: interventricular septum thickness; LVID: left ventricular internal dimension; LVPW: left ventricular posterior wall; LV volume: left ventricular volume; EF: ejection fraction; FS: fractional shortening.

**Table 2:** Echography parameters of wild-type mice with TAC administered either Dex or vehicle

Measurement	Units	Administration groups		*P* value
		Vehicle	Dexmedetomidine	
**IVST**				
diastole	mm	1.02±0.05	1.04±0.06	0.83
systole	mm	1.26±0.09	1.38±0.11	0.43
**LVID**				
diastole	mm	5.03±0.12	4.96±0.13	0.71
systole	mm	4.77±0.15	4.27±0.07	0.034
**LVPW**				
diastole	mm	0.98±0.05	0.98±0.09	0.94
systole	mm	1.03±0.05	1.07±0.07	0.68
**LV volume**				
diastole	μL	120.1±6.9	116.4±6.8	0.71
systole	μL	106.3±7.8	81.9±3.0	0.041
**EF**	%	16.4±2.9	25.5±2.5	0.046
**FS**	%	7.44±1.4	11.9±1.3	0.048

IVST: interventricular septum thickness; LVID: left ventricular internal dimension; LVPW: left ventricular posterior wall; LV volume: left ventricular volume; EF: ejection fraction; FS: fractional shortening.
