# Supplementary material for: An α2-adrenoceptor agonist: Dexmedetomidine induces protective cardiomyocyte hypertrophy through mitochondrial-AMPK pathway
Source: Int J Med Sci. 2020 Sep 9;17(16):2454–67. doi: 10.7150/ijms.47598 (PMC7532472; doi:10.7150/ijms.47598)
Supplement: Supplementary file 1 — Supplementary figures and tables. [file ijmsv17p2454s1.pdf]

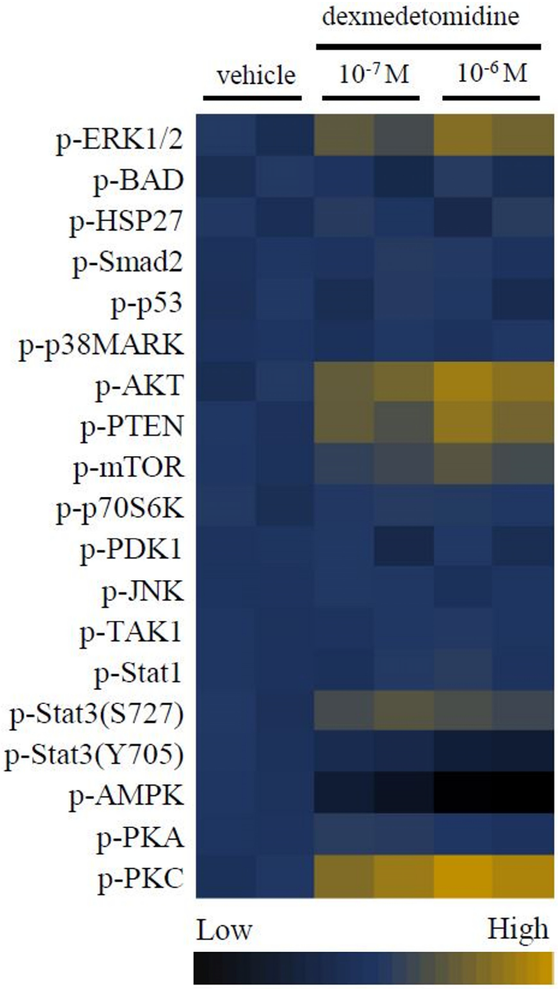

Supplementary Figure 1. Heat map of pathscan intracellular signaling analysis in cardiomyocytes treated with vehicle and two concentrations (10^{-7} and 10^{-6} M) of Dex. Blue represents a low level and yellow represents a relatively high level of expression of each protein.

Supplementary Table 1. Mouse primer sequences used for RT-qPCR.

Gene	Forward sequence (5'-3')	Reverse sequence (5'-3')
Anp	GAGAGACGGCAGTGCTTCTA	CGTGACACACCACAAGGGCTT
Bnp	AGGCGAGACAAGGGAGAACA	GGAGATCCATGCCGCAGA
Myh7	CGGACCTTGGAAGACCAGAT	GACAGCTCCCCATTCTCTGT
Ctgf	CAAAGCAGCTGCAAATACCA	GGCCAAATGTGTCTTCCAGT
Col1	GAGCGGAGAGTACTGGATCGA	CTGACCTGTCTCCATGTTGCA
Col3a	CAACCAGTGCAAGTGACCAA	GCACCATTGAGACATTTTGAAG
Rps 18	TTCTGGCCAACGGTCTAGACA	CCAGTGGTCTTGGTGTGCTGA

Rat primer sequences used for RT-qPCR.

Gene	Forward sequence (5'-3')	Reverse sequence (5'-3')
Anp	GGGGGTAGGATTGACAGGAT	CTCCAGGAGGGTATTCACCA
Bnp	GACGGGCTGAGGTTGTTTTA	ACTGTGGCAAGTTTGTGCTG
Myh7	GAGCCTCCAGAGTTTGCTGAAG	TTGGCACGGACTGCGTCATC
c-jun	TGAAAGCGCAAAACTCCGA	TGTGCCACCTGTTCCCTGA
c-fos	AGTGGTGAAGACCATGTCAGG	CATTGGGGATCTTGCAGG
Rps 18	CGCCGCTAGAGGTGAAATTC	CCAGTCGGCATCGTTTATGG
